# Supplementary material for: 2D quasi-layered material with domino structure
Source: Nat Commun. 2023 Nov 9;14:7225. doi: 10.1038/s41467-023-42818-x (PMC10632391; doi:10.1038/s41467-023-42818-x)
Supplement: Supplementary file 1 — Supplementary Information [file 41467_2023_42818_MOESM1_ESM.pdf]

**Supplementary Information**

# 2D quasi-layered material with domino structure

Lan et al.

## Contents

1. OM images of layered GaTe samples grown on vdW substrate .................................................................. 4

2. Temperature-dependent XRD diagrams of Ga .......................................................................................... 5

3. Calculation of interface interaction between Ga/2D QLDS-GaTe and Ga/layered GaTe ........................ 6

4. TOF-SIMS analysis of Te distribution under varied thickness ................................................................. 7

5. Computed formation energies of different GaTe configurations using vdW growth strategy and ISMG strategy ........................................................................................................................................................ 8

6. Synthesis and growth regulation method for 2D QLDS-GaTe ................................................................. 9

7. OM images of 2D QLDS-GaTe synthesized via ISMG strategy ............................................................ 10

8. OM images of orientated growth 2D QLDS-GaTe crystals ..................................................................... 11

9. OM images of 2D QLDS-GaTe under diverse thicknesses ...................................................................... 12

10. AFM images of 2D QLDS-GaTe under diverse thicknesses .................................................................. 13

11. Structural characterizations of 2D QLDS-$In_4Te_3$ samples .................................................................... 14

12. End-point snapshot from AIMD ............................................................................................................ 15

13. AFM image of 2D QLDS-GaTe crystal .................................................................................................. 16

14. Simulated FFT results of 2D QLDS-GaTe structure with different thicknesses. ................................. 17

15. The EDS elemental mappings of the cross-section of the 2D QLDS-GaTe crystal .............................. 18

16. A HAADF-STEM image of the cross-section of the 2D QLDS-GaTe crystal ....................................... 19

17. Band structure of 2D QLDS-GaTe structure .......................................................................................... 20

18. Depiction of bonding lengths in 2D QLDS-GaTe in comparison to the bulk structure ........................ 21

19. NBO analysis of the inner bonds of the 2D QLDS-GaTe structure ....................................................... 22

20. COHP analysis of bonding states in 2D QLDS-GaTe structure ............................................................ 23

21. NBO analysis of bulk GaTe structure bonds .......................................................................................... 24

22. COHP analysis of bonding states in bulk GaTe structure ...................................................................... 25

23. COHP analysis of surface bonding states in 2D QLDS-GaTe structure ................................................ 26

24. COHP analysis of other surface bonding states in 2D QLDS-GaTe surface ........................................ 27

Supplementary Table 1. Calculated bonding state of the 2D QLDS-GaTe structure corresponding to Supplementary Figure 19 ............................................................................................................................. 28

Supplementary Table 2. Calculated bonding state of the bulk structure corresponding to Supplementary

**Figure 21** ........................................................................................................................................ 29

**Supplementary Table 3. Calculated bonding state of 2D QLDS-GaTe structure corresponding to Supplementary Figure 23** ........................................................................................................ 30

**Supplementary Table 4. Calculated bonding state of 2D QLDS-GaTe structure corresponding to Supplementary Figure 24** ........................................................................................................ 31

## 1. OM images of layered GaTe samples grown on vdW substrate

Previous investigations have demonstrated that van der Waals (vdW) substrates can efficiently assist the growth of layered gallium telluride (GaTe) single crystals[1]. In this growth paradigm, the substrate and material exhibit no mutual interaction. Upon random nucleation of GaTe crystals, their growth proceeds in arbitrary directions, resulting in no structured arrangement. This method, while facilitating growth, fails to deliver large-area GaTe single crystals and proves ineffectual in manipulating the crystal growth orientation.

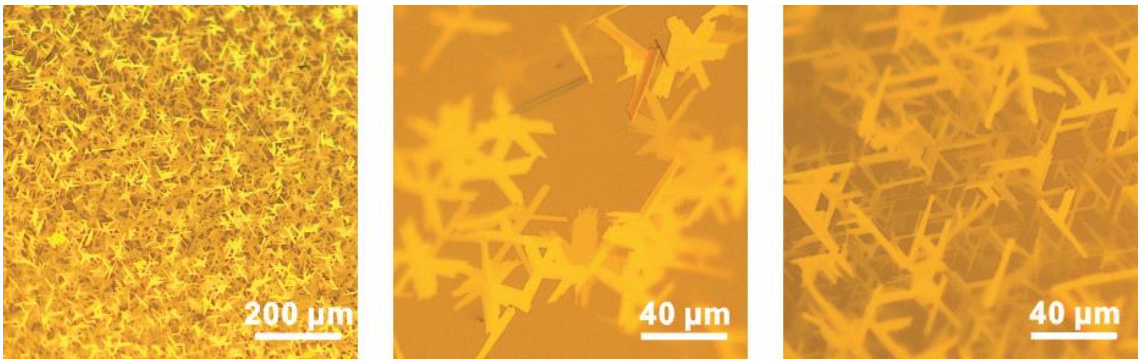


**Supplementary Figure 1 | OM images of layered GaTe samples grown on vdW substrate.**

## 2. Temperature-dependent XRD diagrams of Ga

We conducted X-ray diffraction (XRD) analyses on liquid metal at a range of temperatures. Intriguingly, gallium (Ga), despite its transition to the liquid state, preserves an appreciable degree of ordered structure, predominantly along the (211) plane (ICDD PDF No. 00-031-053). This residual order qualifies the liquid metal as an interactive substrate, capable of directing the growth of GaTe single crystals along predetermined orientations. This finding challenges the established symmetry of forces typically observed in traditional van der Waals growth regimes.

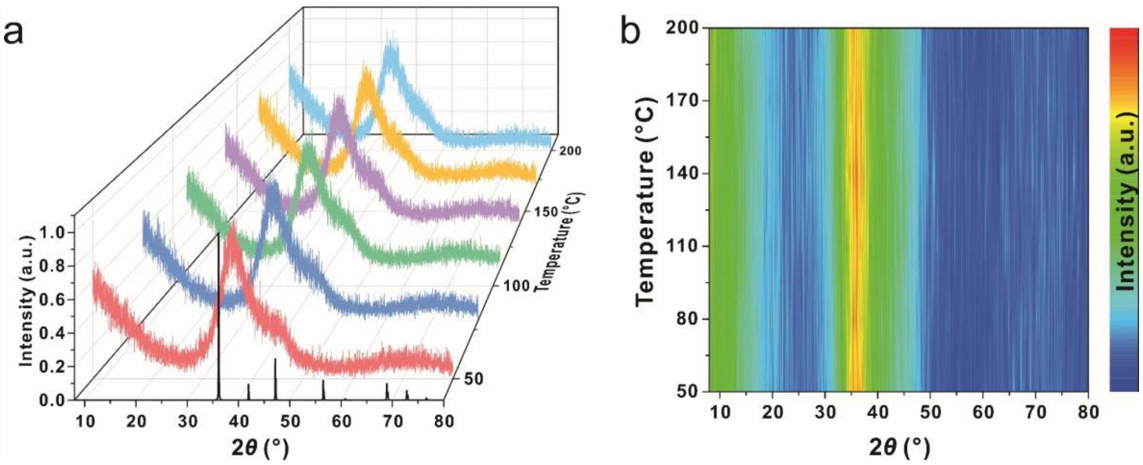


**Supplementary Figure 2 | Temperature-dependent XRD diagrams of Ga. a**, The XRD diagrams of liquid metal under different temperatures. **b**, Projected XRD results extracted from Supplementary Figure 2a.

### 3. Calculation of interface interaction between Ga/2D QLDS-GaTe and Ga/layered GaTe

We established models representing the interaction between liquid metal and 2D quasi-layered domino-structured (QLDS) GaTe, and between liquid metal and layered GaTe. Subsequent density functional theory (DFT) calculations on the strength of these interactions revealed a significantly stronger charge interaction between Ga and 2D QLDS-GaTe (0.0032 e $Å^{-3}$), in stark contrast to that observed with layered GaTe (0.0012 e $Å^{-3}$). This underscores the role of liquid metal Ga in amplifying the longitudinal binding forces with the material, thereby disrupting the material's inherent lateral homogeneity. As a result, the synthesis of 2D QLDS-GaTe becomes a viable possibility.

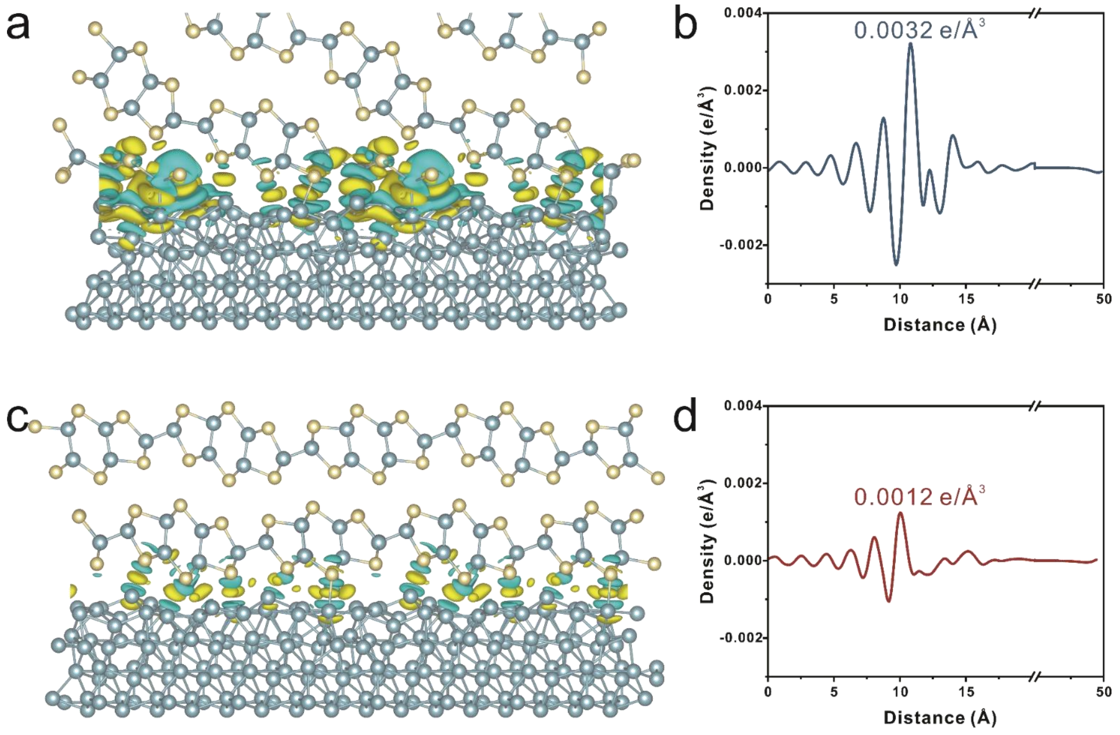


**Supplementary Figure 3 | Interface interaction between Ga/2D QLDS-GaTe and Ga/layered GaTe. a**, **c**, Differential charge density of 2D QLDS-GaTe and layered GaTe on Ga substrate, as calculated via DFT. Areas of electron accumulation and depletion are represented in yellow and cyan, respectively. **b**, **d**, Planar-averaged electron density difference plots corresponding to panels a and c.

## 4. TOF-SIMS analysis of Te distribution under varied thickness

Time-of-Flight Secondary Ion Mass Spectrometry (TOF-SIMS) was employed to probe the spatial distribution of the precursor element Te within liquid Ga, as illustrated in Fig. S4a. Upon precipitation of the 2D QLDS-GaTe at the liquid metal interface, an elevated concentration of Te is observed at the surface, declining towards the bulk phase distal from the interface. An interface double layer-like region is formed, where Te concentration gradually decreases at the junction between the material and the liquid metal. This unique characteristic promotes the crystal growth and facilitates the 2D extension of QLDS-GaTe. Additionally, planar projection results, as shown in Figure S4b, reveal that the synthesized material floats on the liquid metal surface, maintaining a smooth interface which endows the material with excellent mobility on the liquid metal, favoring the growth and assembly into a larger single-crystalline material.

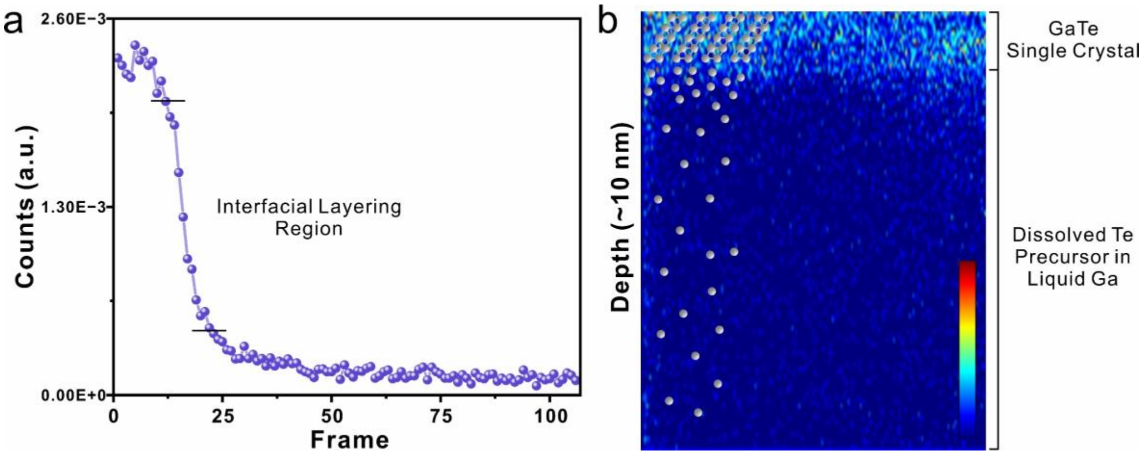


**Supplementary Figure 4 | TOF-SIMS analysis of Te distribution under varied thickness. a**, Graph depicting the concentration distribution of Te as a function of thickness. **b**, Schematic representation of the 2D planar projection of Te element distribution.

**5. Computed formation energies of different GaTe configurations using vdW growth strategy and ISMG strategy**

The computational findings suggest that for the growth of GaTe, the m-phase and h-phase exhibit similar formation energies. Concurrently, the formation energy along the non-planar orientation closely mirrors that of the layered growth mode. This implies that under the vdW growth paradigm, the resulting GaTe may comprise a mixture of phases and polycrystalline material. In contrast, our employed ISMG strategy for GaTe growth can yield 2D QLDS-GaTe across a wide chemical potential window, simultaneously accomplishing phase structure differentiation and orientation segregation. This further validates the viability of the synthesis approach.

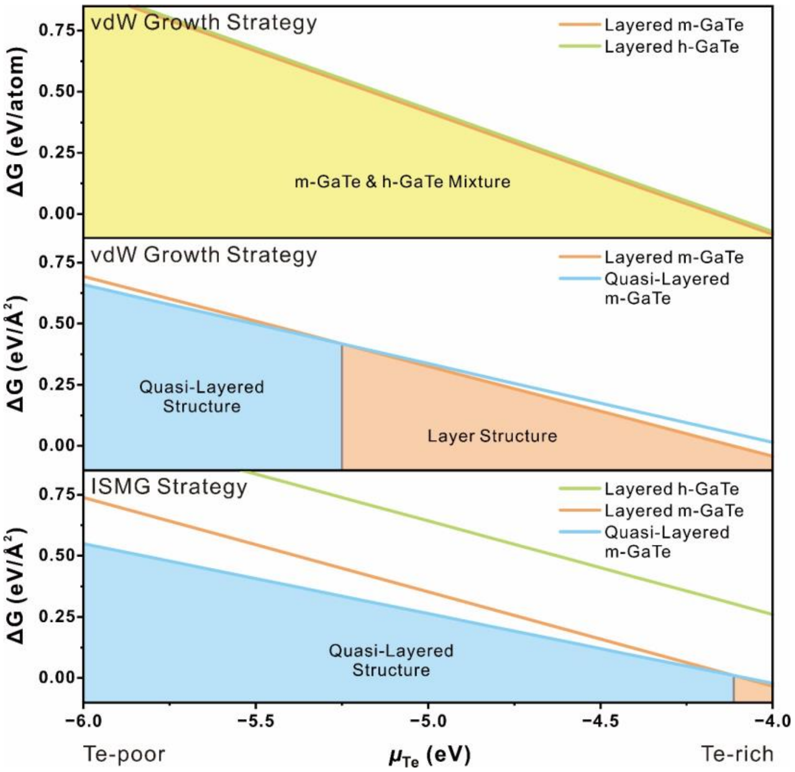


**Supplementary Figure 5 | Computed formation energies of different GaTe configurations using vdW growth strategy and ISMG strategy.**

## 6. Synthesis and growth regulation method for 2D QLDS-GaTe

In the CVD synthesis process of 2D QLDS-GaTe, the liquid metal captures gaseous Te precursors and stores them within its structure. As the cooling process ensues, 2D QLDS-GaTe precipitates on the surface of the liquid metal (Supplementary Fig. 6a, 6b). Consequently, under the same annealing time and varying reaction temperatures, the evaporation rate of the precursor decreases with temperature, leading to a corresponding decline in the amount of Te precursors captured by the liquid metal. Therefore, by modulating the reaction temperature, we can adjust the chemical potential of Te, thereby enabling the synthesis of 2D QLDS-GaTe with different thicknesses.

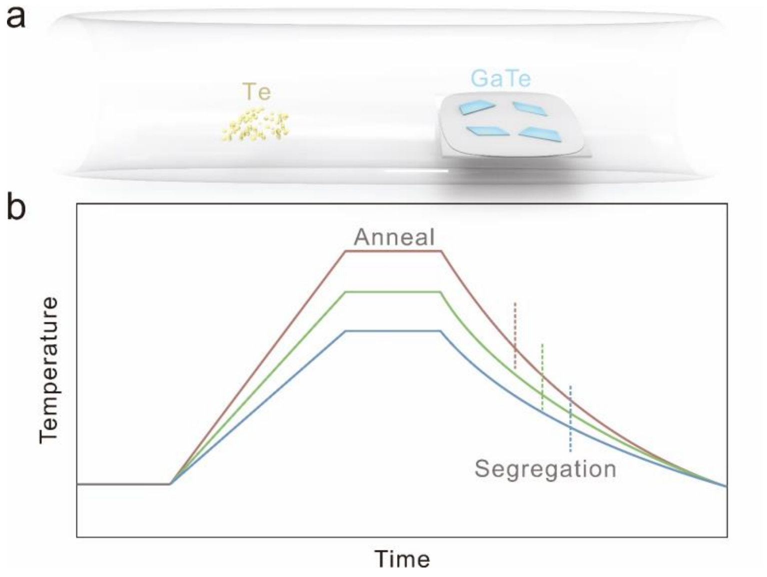


**Supplementary Figure 6 | Synthesis and growth regulation method for 2D QLDS-GaTe. a**, Schematic diagram of 2D QLDS-GaTe synthesis. **b**, Illustrative diagram of annealing processes at different temperatures.

## 7. OM images of 2D QLDS-GaTe synthesized via ISMG strategy

Adopting the ISMG strategy, 2D QLDS-GaTe can be synthesized across a wide chemical potential window. As the chemical potential escalates, the resultant material size also incrementally expands. Given the smooth interface between the liquid metal and 2D QLDS-GaTe, the material can move and coalesce on the surface of the liquid metal, thereby forming a larger scale. As can be observed from the OM images, the 2D QLDS-GaTe exhibits an orderly arrangement. With the ascension of the chemical potential, the grown material can also reach the scale of several hundred micrometers, which is highly conducive to device fabrication. Evidently, the ISMG strategy acts as a vigilant guard, enabling the synthesis of 2D QLDS-GaTe materials of varied sizes and thicknesses at the brink of toppling over.

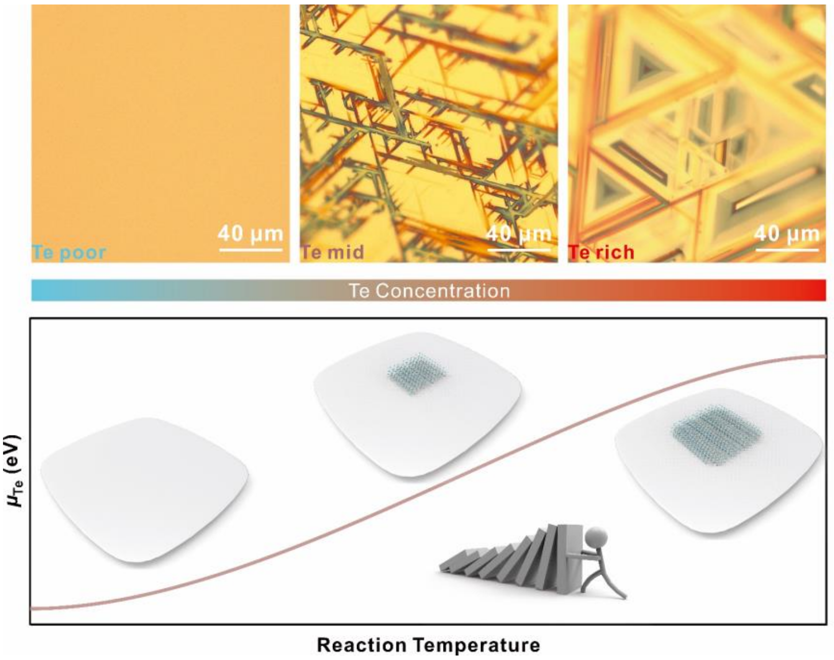


**Supplementary Figure 7 | OM images of 2D QLDS-GaTe synthesized under different chemical potentials guided by ISMG strategy.**

## 8. OM images of orientated growth 2D QLDS-GaTe crystals

Within the framework of the ISMG strategy, liquid metal acts as a template, disrupting inherent forces within the material, and consequently inducing its growth. This directional inducement also prompts a relatively ordered arrangement of the material upon the liquid metal surface. The discovery of this phenomenon has laid a solid foundation for the large-scale synthesis of 2D QLDS-GaTe, offering a promising new avenue for the advancement of 2D material growth strategies.

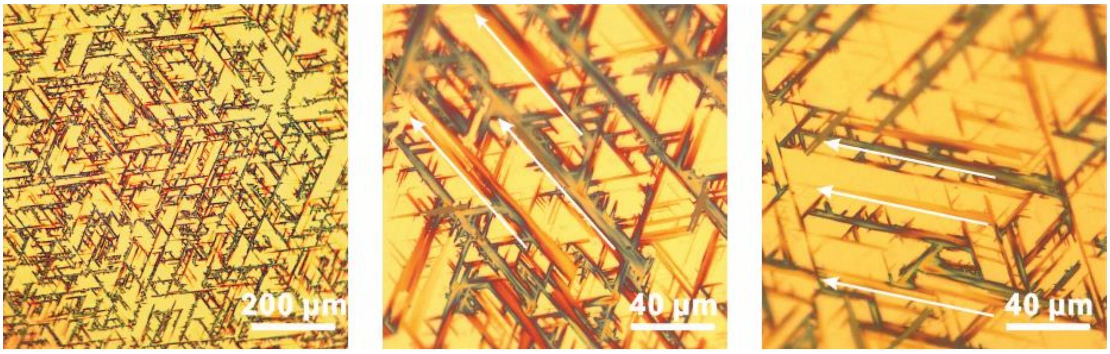


**Supplementary Figure 8 | OM images of orientated growth 2D QLDS-GaTe crystals.**

## 9. OM images of 2D QLDS-GaTe under diverse thicknesses

As the annealing time increases, the amount of Te dissolved in the liquid metal also progressively rises, leading to an elevated chemical potential of Te. During the cooling process, the material tends to precipitate on the surface of the liquid metal. A higher concentration of Te accelerates this precipitation efficiency. While the material continues to exhibit a pronounced anisotropic lateral growth behavior, its vertical growth rate becomes increasingly significant. It's due to this characteristic that we can controllably synthesize 2D QLDS-GaTe of varying thicknesses. Therefore, by modulating the annealing duration, we can achieve the synthesis of a range of 2D QLDS-GaTe with diverse thicknesses.

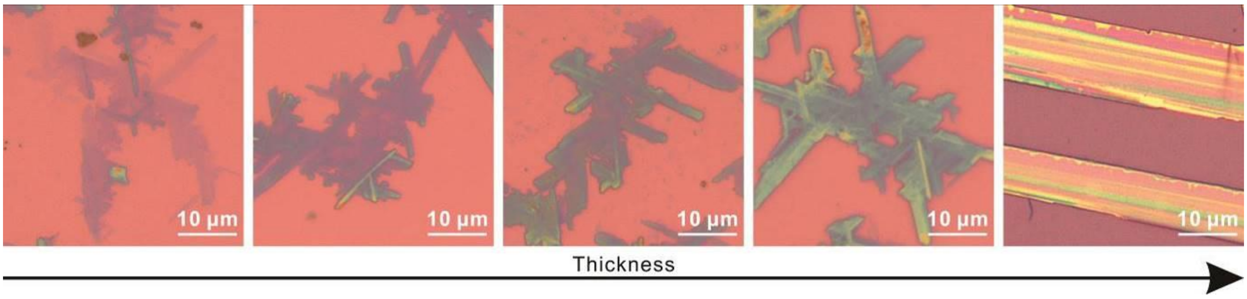


**Supplementary Figure 9 | OM images of 2D QLDS-GaTe under diverse thicknesses.**

## 10. AFM images of 2D QLDS-GaTe under diverse thicknesses

AFM analysis further corroborates that by modulating the annealing duration, we can synthesize 2D QLDS-GaTe samples with a diverse range of thicknesses.

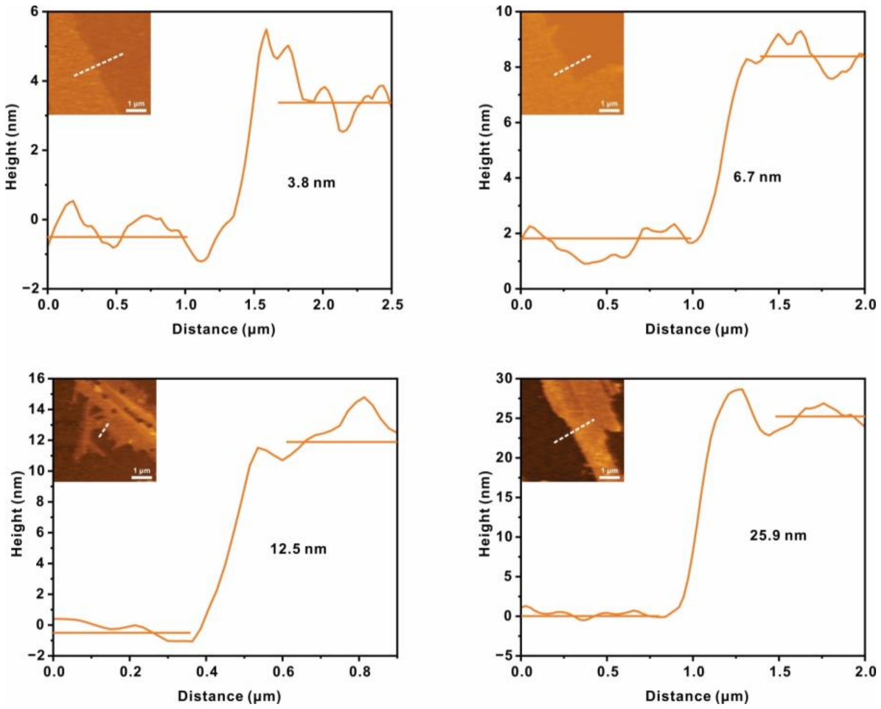


**Supplementary Figure 10 | AFM images of 2D QLDS-GaTe under diverse thicknesses.**

## 11. Structural characterizations of 2D QLDS-$In_4Te_3$ samples

Supplementary Figures 11a,b illuminate the distinct architecture of the material, capturing a notably tilted atomic configuration. The interlayer interactions characterizing this material intriguingly bridge the gap between vdW forces and covalent bonds, precisely resonating with the quintessential traits of QLDS materials. Transitioning to its chemical profile, we harnessed EDS techniques. Evident in Supplementary Figure 11c, the EDS elemental mapping showcases a homogenous In/Te distribution within the $In_4Te_3$ domain, underscoring its compositional consistency. The crystalline integrity of the material is unambiguously manifested in the singular diffraction patterns encapsulated in the FFT visuals in Supplementary Figure 11d, and this assertion gains further traction through the high-resolution TEM image depicted in Supplementary Figure 11e. Herein, prominent lattice spacings, tagged as (200) and (002), reveal a predilection of nascent $In_4Te_3$ samples for growth along the (040) facet, harmonizing with structural narratives in Supplementary Figures 11a–b. These revelations not only vouch for the adaptability of our fabrication approach to a gamut of domino-structured quasi-layered materials but also bolster the edifice for future explorations in this intriguing arena.

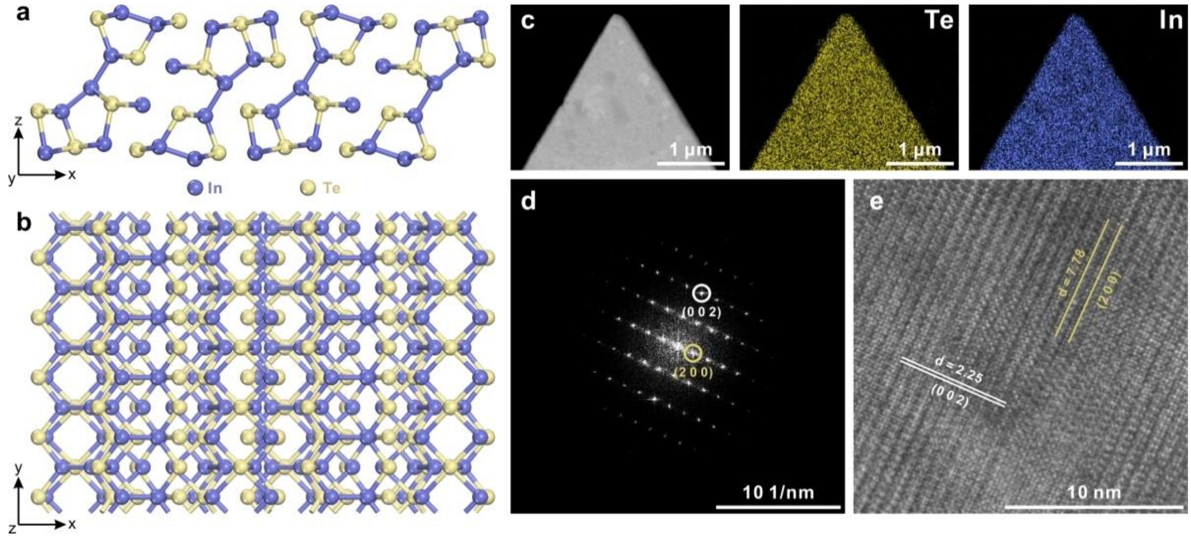


**Supplementary Figure 11 | Structural characterizations of 2D QLDS-$In_4Te_3$ samples. a**, **b**, Side view and top view of 2D QLDS-$In_4Te_3$ structure. **c**, A low magnification TEM image of 2D QLDS-$In_4Te_3$ sample and EDS elemental mappings of Te and In. **d**, **e**, FFT patterns and HRTEM image of 2D QLDS-$In_4Te_3$ sample.

## 12. End-point snapshot from AIMD

To investigate the stability of 2D QLDS-GaTe, ab Initio Molecular Dynamics (AIMD) methods were employed. The system was gradually heated from 100K to 300K over a span of 2 ps, followed by a pre-equilibration period of 10 ps. The results from the subsequent 25 ps were taken as the AIMD simulation outcomes. As evidence, the material maintains a stable crystal structure, thereby verifying its stability at room temperature.

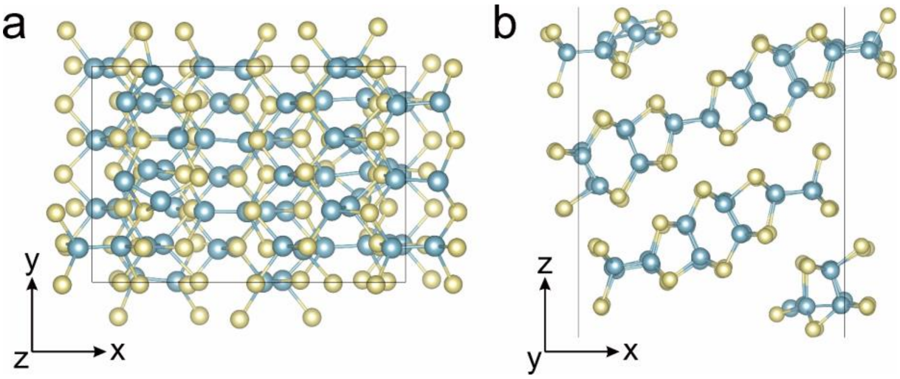


**Supplementary Figure 12 | End-point snapshot from AIMD. a**, **b**, Top and side views of the 2D QLDS-GaTe structure corresponding to Fig. 1f, respectively.

## 13. AFM image of 2D QLDS-GaTe crystal

Atomic Force Microscopy (AFM) results reveal that the synthesized material exhibits a thickness of 1.2 nm, which closely approaches the theoretical limit of the thickness (Fig. 2b). This finding not only validates the feasibility of the ISMG strategy but also paves the way for the manipulation of interlayer interactions in the material. Due to its unique structure, 2D QLDS-GaTe serves as a convenient platform for examining interlayer coupling effects. The experimentally observed contraction of 7.7% is remarkably close to the theoretical limit of 10.8%. This physical phenomenon lays a foundation for acoustic studies under different interlayer coupling conditions, such as thermal conductivity and thermoelectric performance.

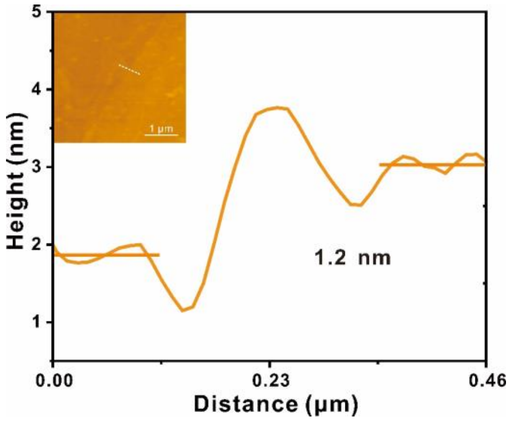


**Supplementary Figure 13 | AFM image of 2D QLDS-GaTe crystal.**

## 14. Simulated FFT results of 2D QLDS-GaTe structure with different thicknesses

As the material thickness diminishes and interlayer coupling effects intensify, the diffraction patterns of 2D QLDS-GaTe correspondingly transform. We simulated the FFT patterns for structures approximately 1 nm and 4 nm thick, derived from our calculations, as shown in the figure below. Given that the lattice constant of the material doesn't exhibit significant variations beyond 2 nm, and the simulated crystal diffraction patterns and interplanar spacings also show no pronounced differences at thicknesses beyond this value, we chose a 4 nm thick model to simulate the diffraction structure characteristic of a bulk crystal. As the interlayer coupling effect intensifies, a significant contraction in the interplanar spacing of the (101) crystal plane becomes apparent, indicating the enhancement of interlayer coupling effects.

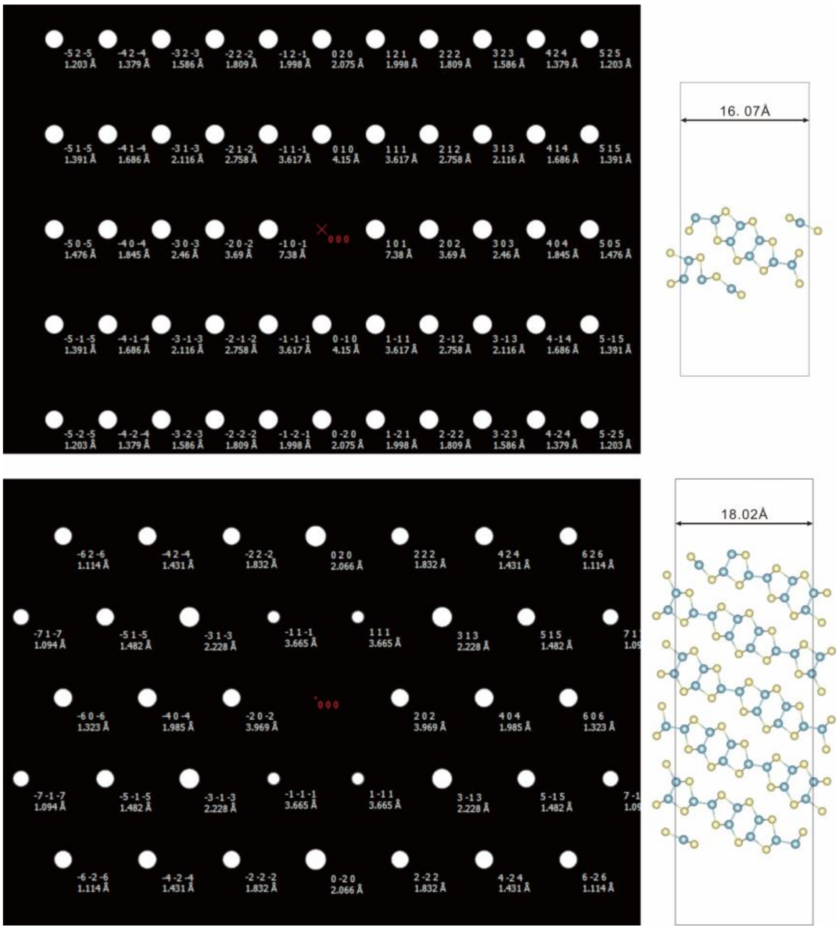


**Supplementary Figure 14 | Simulated FFT results of 2D QLDS-GaTe structure with different thicknesses.**

### 15. The EDS elemental mappings of the cross-section of the 2D QLDS-GaTe crystal

We employed W as a protective layer and conducted cross-sectional EDS elemental mapping on the material. The results revealed a uniform elemental distribution, and the interface with the Si section appeared exceptionally smooth.

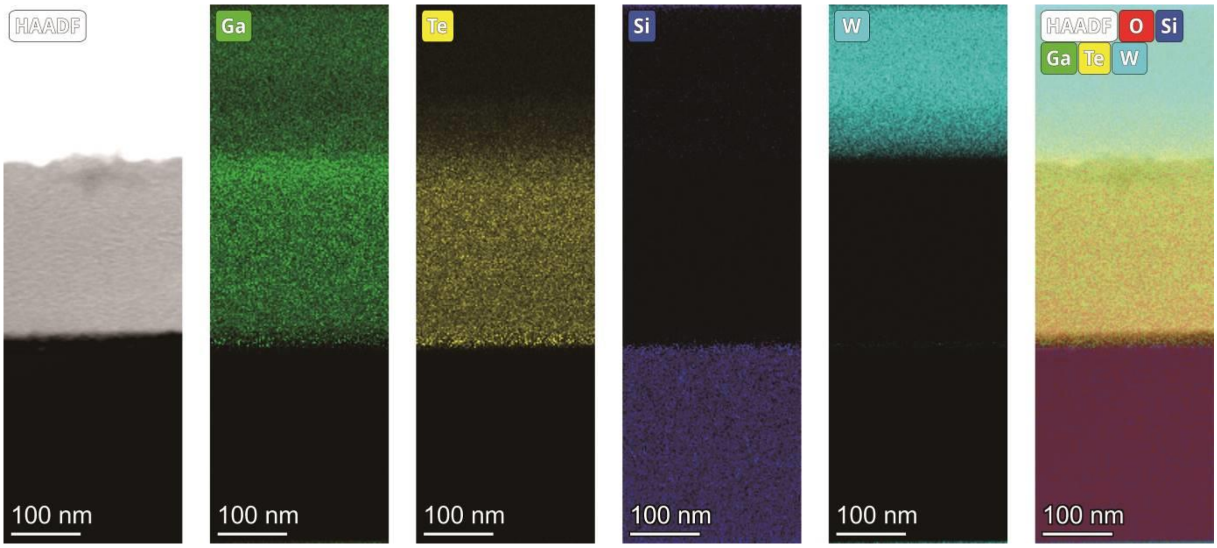


**Supplementary Figure 15 | The EDS elemental mappings of the cross-section of the 2D QLDS-GaTe crystal.**

## 16. A HAADF-STEM image of the cross-section of the 2D QLDS-GaTe crystal

The cross-sectional HADDF-STEM images of the material depict a remarkably smooth interface between 2D QLDS-GaTe and Si. Moreover, there is a notable correspondence with the atomic structure, further substantiating the presence of a domino structure in the derived material.

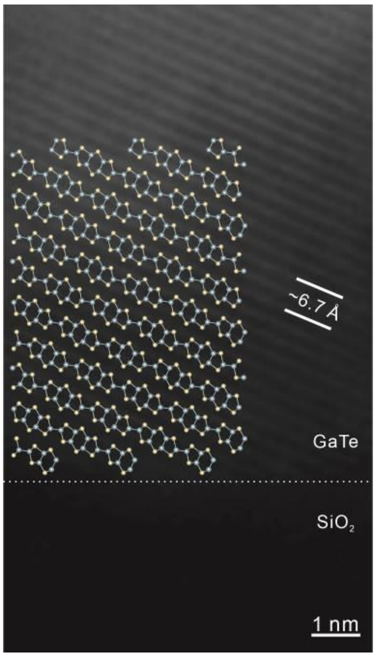


**Supplementary Figure 16 | A HAADF-STEM image of the cross-section of the 2D QLDS-GaTe crystal.**

## 17. Band structure of 2D QLDS-GaTe structure

Distinct from bulk materials, the band structure of 2D QLDS-GaTe exhibits two notable characteristics: 1. A novel charge state emerges at the Fermi energy level, which is provided by surface atoms; 2. Due to quantum confinement effects, the intrinsic bandgap of the material is expanded. These two discoveries suggest promising application prospects for this material in the fields of optoelectronics and catalysis.

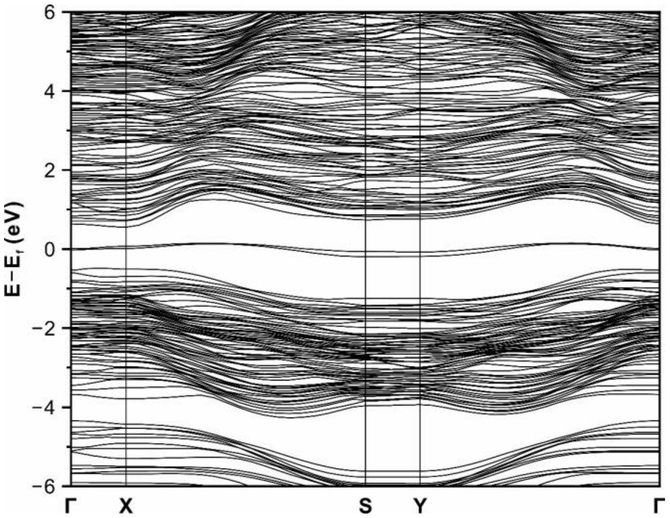


**Supplementary Figure 17 | Band structure of 2D QLDS-GaTe structure.**

## 18. Depiction of bonding lengths in 2D QLDS-GaTe in comparison to the bulk structure

The unsaturated surfaces of 2D QLDS-GaTe prompt surface reconstructions, thereby generating unique bonding states that are distinct from those in bulk materials. For instance, bonds with lengths of ~2.57 Å and ~2.8 Å significantly differ from those in bulk materials. These specialized bonding states endow the material with characteristics that are distinct from layered materials, leading to a further weakening of its symmetry. This is considered one of the reasons for the enhancement of the second harmonic signal.

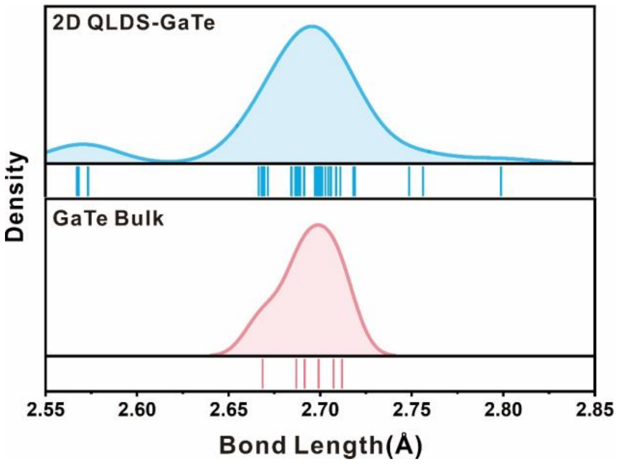


**Supplementary Figure 18 | Depiction of bonding lengths in 2D QLDS-GaTe in comparison to the bulk structure.**

## 19. NBO analysis of the inner bonds of the 2D QLDS-GaTe structure

The Natural Bond Orbital (NBO) results underscore the predominant σ bonding effect between Ga and Te. The bonding orbital is primarily contributed by Te, while the antibonding orbital is attributed to Ga (Table S1). The hybridization of Ga is almost entirely $sp^3$, with the contribution of the d orbital being almost negligible. The hybridizations of Te atoms are $s^{12.10}p^{87.82}d^{0.08}$ and $s^{12.87}p^{87.07}d^{0.06}$, with p orbitals making the primary contribution.

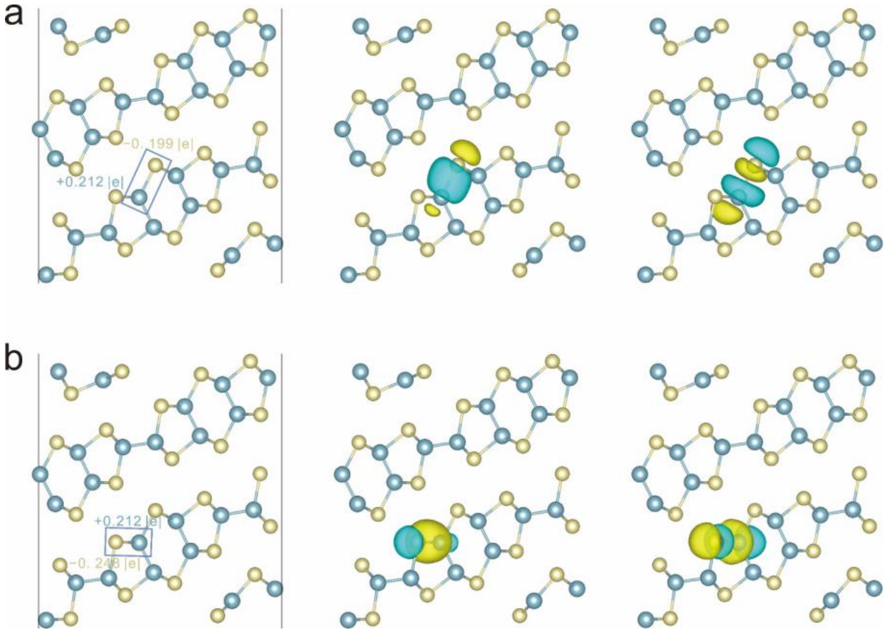


**Supplementary Figure 19 | NBO analysis of the inner bonds of the 2D QLDS-GaTe structure. a**, **b**, Structure and the associated bonding and antibonding orbitals within different chemical environments.

## 20. COHP analysis of bonding states in 2D QLDS-GaTe structure

The Crystal Orbital Hamilton Population (COHP) calculations reveal that the predominant contribution to Ga–Te bonding comes from Ga (4p) and Te (5p) orbitals, followed by substantial contributions from the interactions between Ga(4s) and Te(5p) as well as Ga(4p) and Te(5s). In the subsequent analysis, we will employ these numerical values as benchmarks to determine whether the bonding interactions in the material are consistent.

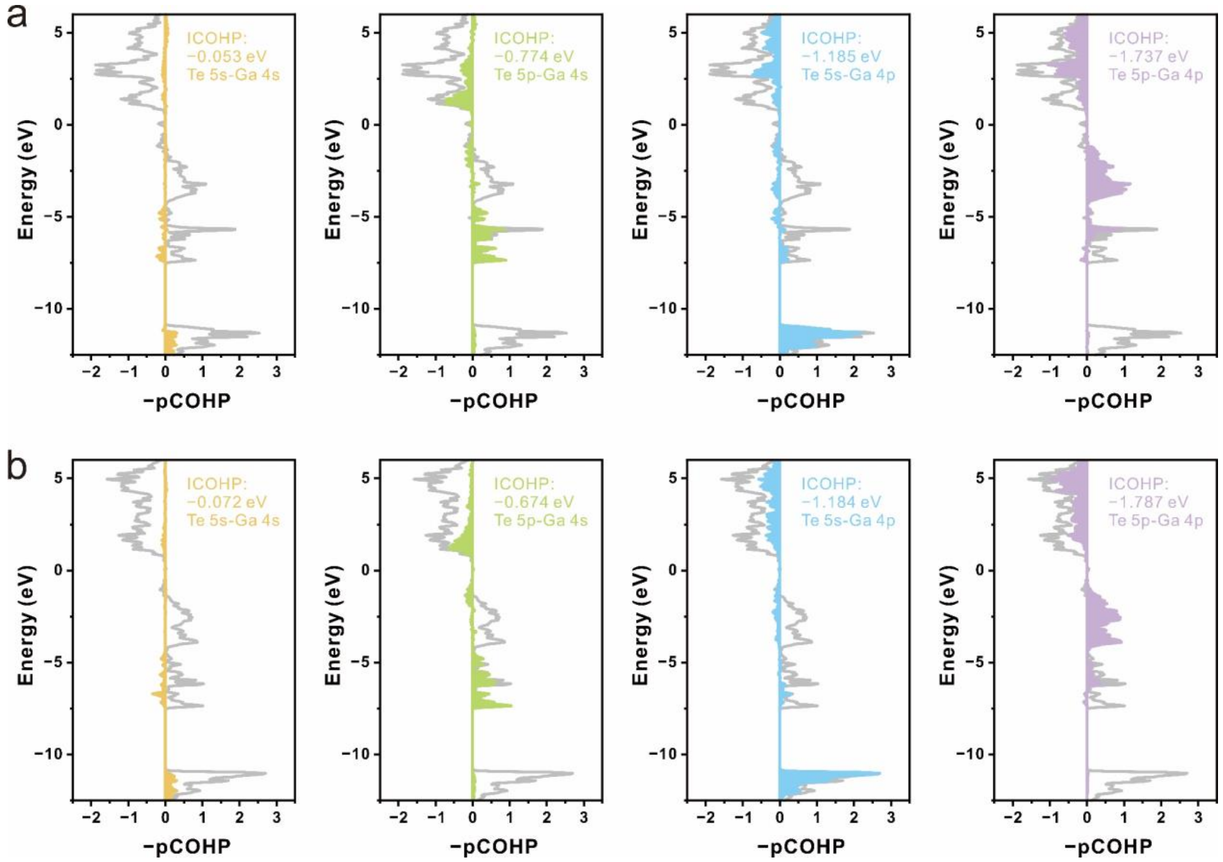


**Supplementary Figure 20 | COHP analysis of bonding states in 2D QLDS-GaTe structure. a**, **b**, COHP diagram for bonds marked in Supplementary Figure 19.

## 21. NBO analysis of bulk GaTe structure bonds

The NBO analysis demonstrates a striking resemblance in the orbital shapes of the inner bonds and bulk structure of 2D QLDS-GaTe. Furthermore, their orbital hybridization, contribution, and electron occupancy exhibit remarkable similarities (Table S2). These findings indicate that the inner atoms in 2D QLDS-GaTe preserve a bonding structure akin to that of the bulk material. These findings indicate that the inner atoms of 2D QLDS-GaTe retain a bonding structure similar to that of the bulk structure.

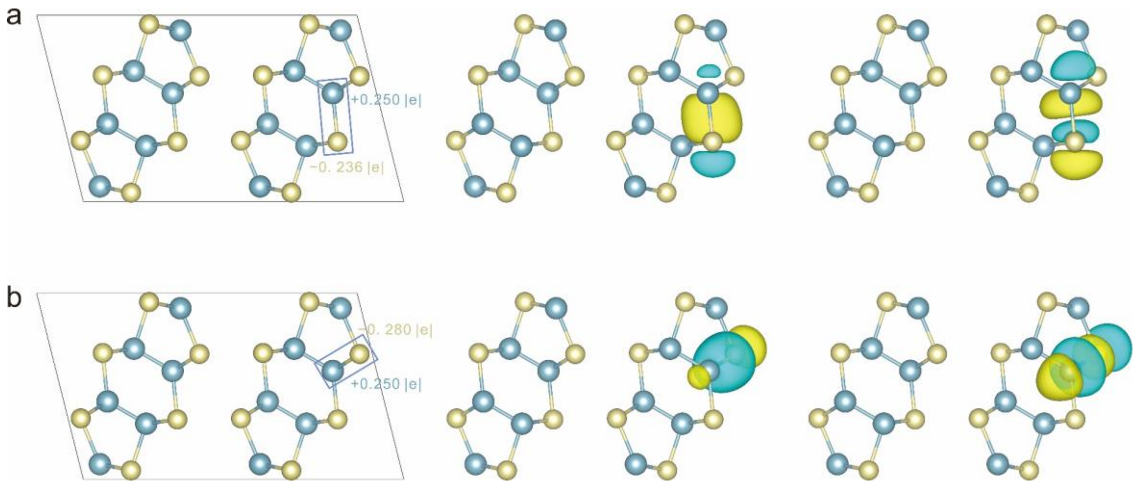


**Supplementary Figure 21 | NBO analysis of bulk GaTe structure bonds. a**, **b**, Representations of the structure and the corresponding bonding and antibonding orbitals within the same bonding environments as depicted in Supplementary Figure 19.

## 22. COHP analysis of bonding states in bulk GaTe structure

From the ICOHP values, it is evident that the corresponding bonding strengths of bulk structure align closely with the inner bonding state of 2D QLDS-GaTe. The COHP plots also exhibit remarkable similarities, further corroborating the resemblance between the two. These observations provide additional evidence for the similarity between the bonding characteristics of the inner bonds and the overall structure of 2D QLDS-GaTe.

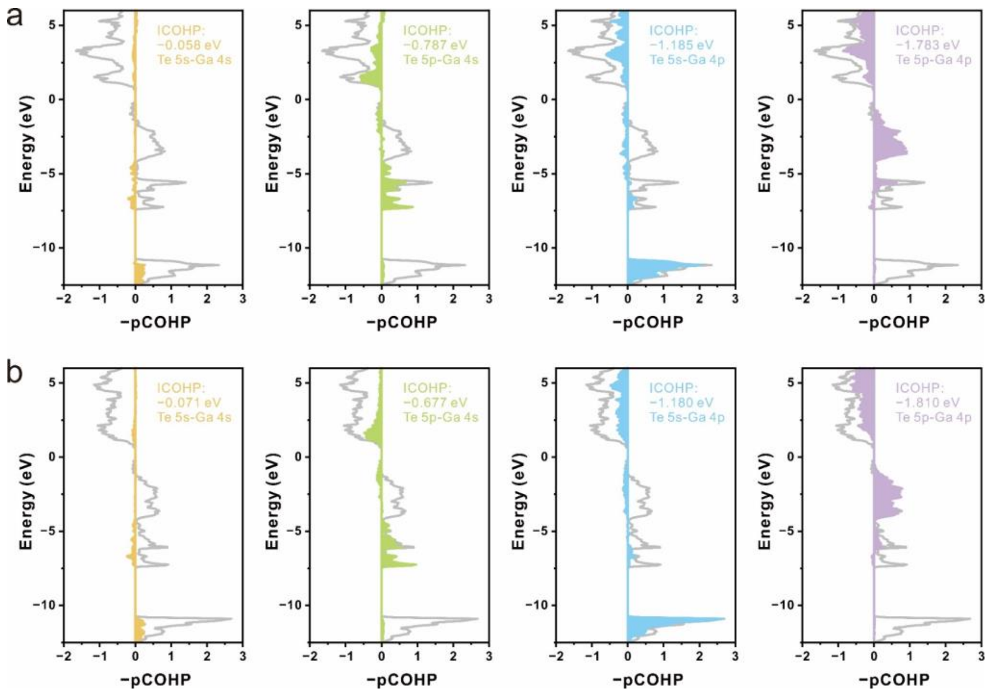


**Supplementary Figure 22 | COHP analysis of bonding states in bulk GaTe structure. a**, **b**, COHP diagram for bonds marked in Supplementary Figure 21.

## 23. COHP analysis of surface bonding states in 2D QLDS-GaTe structure

Based on the values of ICOHP, COHP orbital diagrams, contributions from orbital interactions, and the occupation numbers of orbitals (Supplementary Fig. 23, 24, Table S3, S4), it is evident that the surface bonding states exhibit distinct interactions compared to the inner bonding states. This observation provides insight into the formation of specific bands in the band structure of 2D QLDS-GaTe.

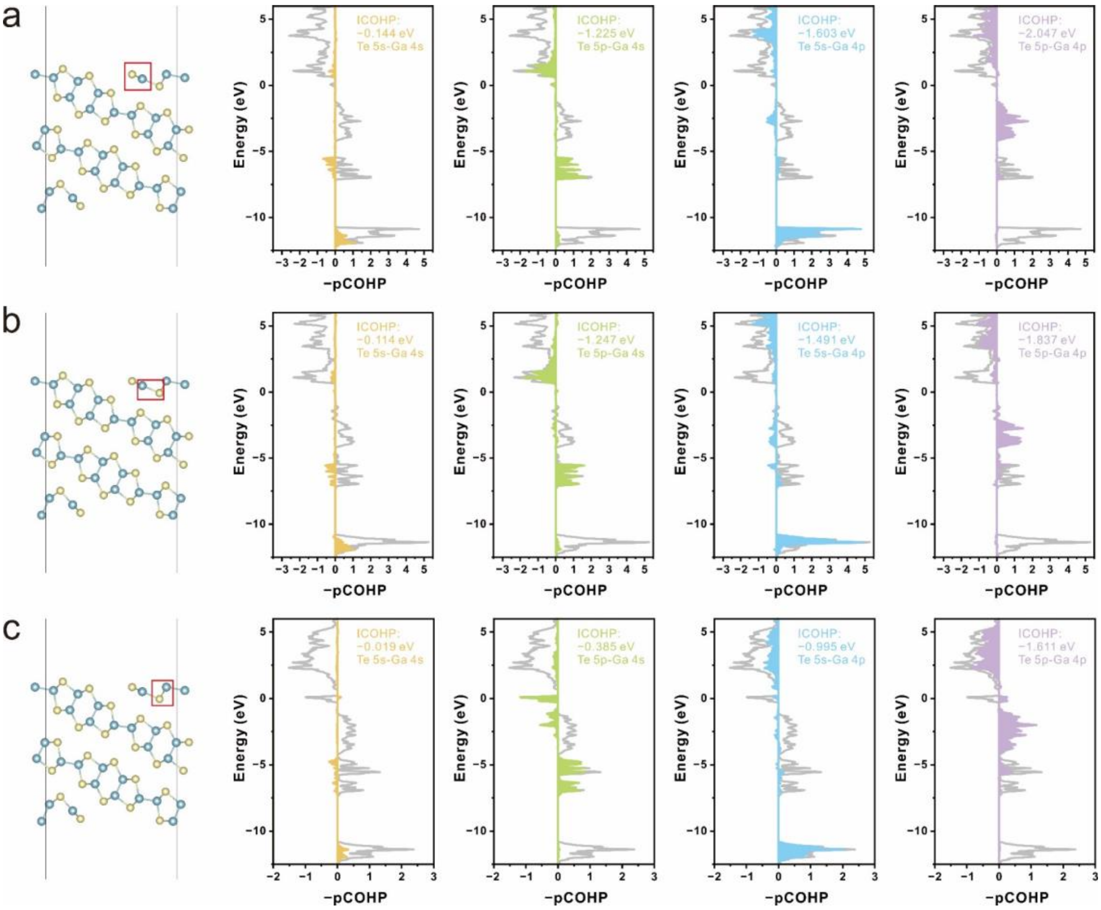


**Supplementary Figure 23 | COHP analysis of surface bonding states in 2D QLDS-GaTe surface.** Panels (a), (b), and (c) illustrate three representative bonding states formed through surface reconstruction.

## 24. COHP analysis of other surface bonding states in 2D QLDS-GaTe surface

We also consider another possible surface. It exhibits the relatively same result mentioned in Supplementary Figure 23. Therefore, the proportions of surface charge states within the system play a crucial role in determining the band structure, thus further influencing the electronic properties. As the thickness of the material decreases, significant changes occur in the band structure of 2D QLDS-GaTe. This alteration in the band structure accounts for the enhanced intensity of second harmonic generation (SHG). Additionally, these findings shed light on its potential applications in nonlinear optics.

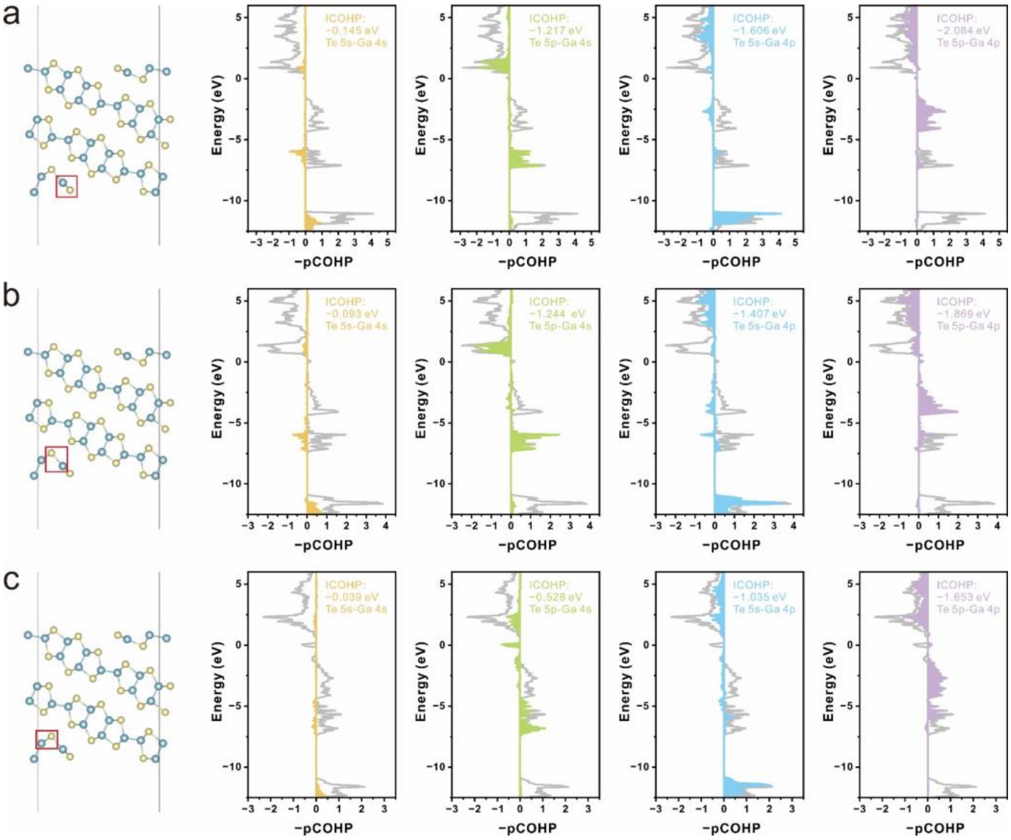


**Supplementary Figure 24 | COHP analysis of other surface bonding states in 2D QLDS-GaTe surface. a**, **b**, **c**, Representative bonding states on another surface.

**Supplementary Table 1. Calculated bonding state of the 2D QLDS-GaTe structure corresponding to Supplementary Figure 19**

| NBO | Occupancy | Center (bond contribution, %) | | Hybridization (Function, %) | |
|---|---|---|---|---|---|
| **Bond1 (σ)** | 1.902 | Ga (27.89) | Te (72.11) | Ga ($s^{25.10}p^{74.90}$) | Te ($s^{12.03}p^{87.97}$) |
| **Bond1 (σ*)** | 0.143 | Ga (72.11) | Te (27.89) | Ga ($s^{25.10}p^{74.90}$) | Te ($s^{12.03}p^{87.97}$) |
| **Bond2 (σ)** | 1.910 | Ga (25.98) | Te (74.02) | Ga ($s^{19.92}p^{80.08}$) | Te ($s^{12.94}p^{87.06}$) |
| **Bond2 (σ*)** | 0.116 | Ga (74.02) | Te (25.98) | Ga ($s^{19.92}p^{80.08}$) | Te ($s^{12.94}p^{87.06}$) |

**Supplementary Table 2. Calculated bonding state of the bulk structure corresponding to Supplementary Figure 21**

| NBO | Occupancy | Center (bond contribution, %) | | Hybridization (Function, %) | |
|---|---|---|---|---|---|
| **Bond1 (σ)** | 1.904 | Ga (27.78) | Te (72.22) | Ga ($s^{25.11}p^{74.72}d^{0.17}$) | Te ($s^{12.10}p^{87.82}d^{0.08}$) |
| **Bond1 (σ*)** | 0.144 | Ga (72.22) | Te (27.78) | Ga ($s^{25.11}p^{74.72}d^{0.17}$) | Te ($s^{12.10}p^{87.82}d^{0.08}$) |
| **Bond2 (σ)** | 1.910 | Ga (25.68) | Te (74.32) | Ga ($s^{20.02}p^{79.86}d^{0.12}$) | Te ($s^{12.87}p^{87.07}d^{0.06}$) |
| **Bond2 (σ*)** | 0.120 | Ga (74.32) | Te (25.68) | Ga ($s^{20.02}p^{79.86}d^{0.12}$) | Te ($s^{12.87}p^{87.07}d^{0.06}$) |

**Supplementary Table 3. Calculated bonding state of 2D QLDS-GaTe structure corresponding to Supplementary Figure 23**

| NBO | Occupancy | Center (bond contribution, %) | | Hybridization (Function, %) | |
|---|---|---|---|---|---|
| **Bond1 (σ)** | 1.907 | Ga (31.83) | Te (68.17) | Ga ($s^{32.78}p^{67.22}$) | Te ($s^{12.07}p^{87.93}$) |
| **Bond1 (σ*)** | 0.129 | Ga (68.17) | Te (31.83) | Ga ($s^{32.78}p^{67.22}$) | Te ($s^{12.07}p^{87.93}$) |
| **Bond2 (σ)** | 1.910 | Ga (31.43) | Te (68.57) | Ga ($s^{34.55}p^{65.45}$) | Te ($s^{10.78}p^{89.22}$) |
| **Bond2 (σ*)** | 0.116 | Ga (68.57) | Te (31.43) | Ga ($s^{34.55}p^{65.45}$) | Te ($s^{10.78}p^{89.22}$) |
| **Bond3 (σ)** | 1.731 | Ga (17.38) | Te (82.62) | Ga ($s^{33.21}p^{66.79}$) | Te ($s^{14.00}p^{86.00}$) |
| **Bond3 (σ*)** | 0.549 | Ga (82.62) | Te (17.38) | Ga ($s^{33.21}p^{66.79}$) | Te ($s^{14.00}p^{86.00}$) |

**Supplementary Table 4. Calculated bonding state of 2D QLDS-GaTe structure corresponding to Supplementary Figure 24**

| NBO | Occupancy | Center (bond contribution, %) | | Hybridization (Function, %) | |
|---|---|---|---|---|---|
| **Bond1 (σ)** | 1.907 | Ga (31.67) | Te (68.33) | Ga ($s^{32.42}p^{67.58}$) | Te ($s^{12.40}p^{87.60}$) |
| **Bond1 (σ*)** | 0.123 | Ga (68.33) | Te (31.67) | Ga ($s^{32.42}p^{67.58}$) | Te ($s^{12.40}p^{87.60}$) |
| **Bond2 (σ)** | 1.885 | Ga (31.34) | Te (68.66) | Ga ($s^{35.30}p^{64.70}$) | Te ($s^{6.75}p^{93.25}$) |
| **Bond2 (σ*)** | 0.180 | Ga (68.66) | Te (31.34) | Ga ($s^{35.30}p^{64.70}$) | Te ($s^{6.75}p^{93.25}$) |
| **Bond3 (σ)** | 1.900 | Ga (23.66) | Te (76.34) | Ga ($s^{22.94}p^{77.06}$) | Te ($s^{11.06}p^{88.94}$) |
| **Bond3 (σ*)** | 0.199 | Ga (76.34) | Te (23.66) | Ga ($s^{22.94}p^{77.06}$) | Te ($s^{11.06}p^{88.94}$) |

## Reference

1. Zallo, E. *et al.* Two-dimensional single crystal monoclinic gallium telluride on silicon substrate via transformation of epitaxial hexagonal phase. *npj 2D Mater. Appl.* **7**, 19 (2023).
